# Haemorrhoids among Patients Visiting the Department of Surgery in a Tertiary Care Military Hospital of Nepal: An Observational Study

**DOI:** 10.31729/jnma.8824

**Published:** 2024-11-30

**Authors:** Chiran Bhakta Bista, Suchit Thapa Chhetri, Bishal Kunwor, Sumit Kumar Sah, Tekendra Adhikari, Nilam Kafle, Dhiran Gurung, Priyanka Tamang, Prem Khadka Thyayat, Nitesh Kumar Shah

**Affiliations:** 1 Department of Surgery, Shree Birendra Hospital, Chhauni, Kathmandu, Nepal; 2 Nepalese Army Institute of Health Sciences, Sanobharyang, Kathmandu, Nepal

**Keywords:** *constipation*, *haemorrhoids*, *prevalence*, *rectal diseases*, *risk factors*

## Abstract

**Introduction:**

Haemorrhoids is a common issue in the anorectal region and involves venous engorgement in the region. It greatly diminishes the overall quality of life of affected individuals by disrupting their physical and mental health. This descriptive cross-sectional study aimed to ascertain the prevalence of haemorrhoids and the associated risk factors among patients visiting a tertiary care center's surgery department.

**Methods:**

A descriptive cross-sectional study was conducted in the Department of Surgery of a tertiary care center from July 2023 to November 2023 after obtaining ethical approval. Convenience sampling method was used. The total sample size included in the study was 385. The data was analyzed in IBM SPSS Statistics software. Point estimate was calculated at 95% confidence interval.

**Results:**

Among 385 patients included in the study, haemorrhoids was prevalent in 53 (13.76%, 95% CI: 8.76%-18.76%). Internal haemorrhoids was most prevalent with 30 (56.60%) cases. 22 (41.51%) cases presented with Grade 1 haemorrhoids. The most prevalent symptoms were bleeding in 29 (54.72%) cases, perianal pain in 25 (47.17%) cases, itching in 28 (33.96%) cases and burning sensation in 15 (28.30%) cases.

**Conclusions:**

The prevalence of haemorrhoids in our center was found to be higher as compared to similar studies done in Southeast Asian region but similar with studies done in other region.

## INTRODUCTION

Haemorrhoids are defined as the engorgement of the venous tissue in the anal region.^1^ Haemorrhoids, a common issue in the anorectal region, greatly disrupt the daily life of those affected both physically and mentally, leading to discomfort, frequent reoccurrence, incomplete relief, and postoperative pain, all of which diminish overall quality of life.^2^

A study conducted in Nepal showed higher prevalence of anorectal disorder among which haemorrhoids was most prevalent, constituting 31.20%.^3^ The prevalence of Haemorrhoids and other anorectal disorders are found to be higher in general population than seen in our clinical settings.^4^ In our society, there's a social stigma attached to haemorrhoids, leading affected individuals to often disregard their issues and thus the condition remains underestimated and underdiagnosed.^5,6^

This descriptive cross-sectional study was conducted with the objective of determining the prevalence of haemorrhoids and their associated risk factors among patients visiting the surgery department of a tertiary care centre.

## METHODS

A descriptive cross-sectional study was conducted prospectively among patients visiting the Department of Surgery in Shree Birendra Hospital after obtaining ethical approval from the Institutional Review Committee of Nepalese Army Institute of Health Sciences (NAIHS-IRC Registration number: 863). The patients visiting surgery department and above 18 years of age were included after obtaining written informed consent from July 7^th^, 2023 to November 6th, 2023. The study excluded patients who were severely ill, mentally ill, or unable to communicate. A convenience sampling method was used. The sample size was calculated using the following formula:


$$\begin{array}{ll} \text{n} & =\frac{{\text{(Z}}^{2}\times \text{p}\times \text{q)}}{{\text{e}}^{\text{2}}}\\ & =\frac{(1.96^{2}\times \text{0.5}\times \text{0.5)}}{0.05^{2}}\\ & =385 \end{array}$$

Where,

n = minimum required sample size
z = 1.96 at 95% Confidence Interval (CI)
p = prevalence of haemorrhoids taken as 50% to obtain maximum sample size
q = 1-p
e = margin of error, 5%

The calculated minimum sample size was 385. The proforma included demographic profile, clinical characteristics and behavioral characteristics which were filled up by interviewing with the patient and observing from their medical book.

Haemorrhoids are diagnosed based on history and anorectal examination which includes inspection, digital rectal examination and anoscope.^7^ Haemorrhoids are usually categorized into internal and external types based on their anatomical location. Internal haemorrhoids originate above the dentate line and are enveloped by columnar epithelium, whereas external haemorrhoids emerge below the dentate line and are covered by squamous epithelium.^8^ Additionally, haemorrhoids are graded into four categories: Grade I, which do not prolapse and appear as a bulge into the lumen of the anal canal; Grade II, which prolapse but spontaneously reduce; Grade III, which prolapse and require manual repositioning; and Grade IV, where the prolapse remains outside of the anus and is irreducible.^7,8^

Data was entered and analyzed in IBM SPSS Statistics. The point estimate was calculated at a 95% confidence interval. Frequency and percentage analysis was performed for the categorical variables.

## RESULTS

Out of 385 patients, 53 (13.76%) (8.76-18.76, 95% Confidence Interval) individuals were diagnosed with haemorrhoids. 31 (58.00%) of haemorrhoid cases were observed in the age group of 30-60 years. The predominant demographics included males, accounting for 31 (58.49%) cases, urban residents totaling 35 (58.49%) cases, and individuals identifying as Hindu by religion, constituting 42 (79.25%) cases. Secondary education was the prevalent educational background, noted in 17 (32.08%) cases, followed closely by primary education, with 16 (30.19%) cases. Additionally, 27 (50.94%) cases of haemorrhoids reported an income range of 20000-40000 (Table 1).

**Table 1:** Demographic Profile (N=385).

Category	Total participants (N=385)	Participants with hemorrhoids n(%)
**Age**		
<30	85 (22.08)	6 (11.32)
30-60	194 (50.39)	31 (58.00)
>60	106 (27.53)	16 (29.60)
**Sex**		
Male	225 (58.44)	31 (58.49)
Female	160 (41.56)	22 (41.51)
**Residence**		
Urban	269 (69.87)	35 (66.04)
Rural	116 (30.13)	18 (33.96)
**Religion**		
Hindu	324 (84.16)	42 (79.25)
Buddhist	15 (3.90)	3 (5.66)
Christian	46 (11.95)	8 (15.09)
**Education**		
Primary	124 (32.21)	16 (30.19)
Secondary	153 (39.74)	17 (32.08)
Higher Secondary	57 (14.81)	9 (16.98)
Graduated	35 (9.09)	1 (1.89)
Uneducated	16 (4.16)	10 (18.87)
**Income**		
<20000	151 (39.22)	18 (33.96)
20000-40000	173 (44.94)	27 (50.94)
40000-60000	49 (12.73)	7 (13.21)
>60000	12 (3.12)	1 (1.89)

The majority of haemorrhoids observed were of the internal type, with a frequency of 30 (56.60%), and were graded as Grade 1 in 22 (41.51%) cases. Among patients with haemorrhoids, the prevalent symptoms included bleeding in 29 (54.72%) cases, perianal pain in 25 (47.17%) cases, itching in 18 (33.96%) cases, and a burning sensation in 15 (28.30%) cases. The majority of patients reported a duration of symptoms exceeding two years, constituting 17 (32.08%) cases, while 16 (30.19%) cases reported symptoms lasting less than six months (Table 2).

**Table 2:** Clinical Characteristics of Haemorrhoids (N=53).

Variable	n (%)
**Classification of Hemorrhoids**	
External Haemorrhoids	23 (43.40)
Internal Haemorrhoids	30 (56.60)
**Classification of Hemorrhoids**	
Grade 1	22 (41.51)
Grade 2	17 (32.08)
Grade 3	12 (22.64)
Grade 4	2 (3.77)
Symptoms^*^	
Bleeding	29 (54.72)
Perianal Pain	25 (47.17)
Itching	18 (33.96)
Burning Sensation	15 (28.30)
**Duration of Symptoms**	
<6 Months	16 (30.19)
6 Months - 1 Year	10 (18.87)
1 Year - 2 Year	10 (18.87)
> 2 Year	17 (32.08)

* Multiple responses

There were 44 (83.02%) patients with haemorrhoids reported consuming a mixed diet, while physical exercise was undertaken by 34 (64.15%) cases, 46 (86.79%) cases adhere to a fiber-rich, 29 (54.72%) patients had experienced constipation, while 12 (22.64%) patients had history of chronic/repeated diarrhea. They occasionally consume fast food, observed in 28 (52.83%) cases. Among females with haemorrhoids, multiparous individuals were prevalent, accounting for 11 (20.75%). Furthermore, there was mostly no reported family history of haemorrhoids, with 35 (66.04%) cases (Table 3).

**Table 3:** Behavioural Characteristics of Haemorrhoids (N= 53).

Variable	n (%)
**Dietary Habit**	
Vegetarian	9 (16.98)
Mixed	44 (83.02)
**Physical Exercise**	
Yes	34 (64.15)
No	19 (35.85)
**Smoking**	
No	23 (43.40)
Previous History	18 (33.96)
Currently Smoking	12 (22.64)
**Alcohol**	
No	21 (39.62)
Previous History	23 (43.40)
Current Intake	9 (16.98)
**Fiber Diet Intake**	
Yes	46 (86.79)
No	7 (13.21)
**Constipation**	
Yes	29 (54.72)
No	24 (45.28)
**Fast Food Consumption**	
Everyday	12 (22.64)
Every alternate day	10 (18.87)
Once a week	3 (5.66)
Occasionally	28 (52.83)
History of Chronic/Repeated Diarrhea	18 (33.96)
Yes	12 (22.64)
No	41 (77.36)
**Parity (For Female)**	
Nulliparous	5 (9.43)
Primiparous	6 (11.32)
Multiparous	11 (20.75)
**Body Mass Index (BMI)**	
Underweight (<18.5)	2 (3.77)
Normal (18.5-24.9)	31 (58.49)
Overweight (25-29.9)	11 (20.75)
Obese (30-34.9)	8 (15.09)
Extremely Obese (>35)	1 (1.89)
Family History of Haemorrhoids	41 (77.36)
Yes	18 (33.96)
No	35 (66.04)

## DISCUSSION

Out of 385 patients, the prevalence of haemorrhoids was seen in 53 (13.76%) patients. This prevalence rate is notably higher than that reported in a similar study conducted in similar setting in South India, where the prevalence was found to be 9.08%.^9^ Riss et al. reported a substantially higher prevalence of haemorrhoids at 39.93% compared to our findings.^10^ Kibret et al. observed a prevalence of 13.1% in their study in Ethiopia.^2^ Al-Masoudi et al. found a prevalence of 16% in their study in Saudi Arabia.^11^ Another study in Saudi Arabia by Oberi et al. revealed that 59% of individuals exhibited at least one hemorrhoidal symptom.^12^ Disparities in prevalence across different countries may be due to variances in lifestyle patterns and health-seeking behaviours of the people, and the availability of healthcare facilities.

In our study, 58% patients diagnosed with haemorrhoids fell within the age group of 30-60 years. This is similar to the findings from a study conducted in India, where 68.05% of patients diagnosed with haemorrhoids were also within the 31-60 age bracket.^9^ Our results align with numerous other studies where the highest prevalence of haemorrhoids is observed within the 30 to 60 age range.^2,11,13^ Additionally, our study revealed that 58.49% of diagnosed cases were males, while 41.51% were females. This gender distribution is consistent with findings from other studies conducted in Southeast Asia, where males tend to outnumber females.^9,13-15^ However, in studies conducted outside of Southeast Asia, there are instances where females outnumber males.^2,12^ The elevated incidence of male cases in the Southeast Asian region might be due to the stigma surrounding this ailment, as well as the reluctance and shy nature of females in discussing and addressing anorectal conditions and undergoing anal examinations for haemorrhoid diagnosis.

In our study, 41.51% of patients were found to have Grade 1 haemorrhoids, followed by Grade 2, Grade 3, and Grade 4, mirroring findings from studies by Riss et al.^10^ and Kibret et al.,^2^ where the majority of patients were diagnosed with Grade 1, followed by Grade 2, Grade 3, and Grade 4. However, in studies by Ponkiya et al.^13^ and Khan et al.,^14^ the majority of patients were diagnosed with Grade III haemorrhoids. Among patients diagnosed with haemorrhoids in our study, 54.72% experienced symptoms of bleeding, while 47.17% reported perianal pain, which are the most commonly associated symptoms with haemorrhoids. Numerous studies have also reported bleeding and perianal pain as the most prevalent symptoms of haemorrhoids.^11-13^ In our study, 32.08% of patients exhibited symptoms of haemorrhoids for more than 2 years, a finding similar to that reported by Najar et al.,^9^ where over 65% of patients experienced symptoms for more than one year. The prolonged duration of symptoms may be due to individuals' reluctance, stemming from shyness and hesitation, to discuss symptoms initially, with many seeking medical advice for haemorrhoids only when symptoms worsen.

Our study revealed that 83.02% of patients diagnosed with haemorrhoids had a mixed diet, and 54.72% of patients also experienced constipation. Similar associations between consuming a mixed diet and experiencing constipation alongside haemorrhoids have been identified in various studies.^2,9,13,14^ Mixed diets typically lack sufficient fiber and prolong bowel transit time, thereby elevating the risk of constipation. Constipation, in turn, can contribute to haemorrhoids by causing the passage of hard stool and increasing intra-abdominal pressure, leading to engorgement of the hemorrhoidal plexus.^1^

While 58.49% of cases in our study exhibited a normal BMI, an increase in BMI has been identified as a significant risk factor for haemorrhoids in previous studies.^2,10^ Additionally, 33.96% of individuals diagnosed with haemorrhoids had a positive family history, aligning with findings from the study by Al- Masoudi et al.,^11^ which also demonstrated a significant association between family history and haemorrhoids. Furthermore, multiparous women were found to have a higher risk of developing haemorrhoids compared to nulliparous women, consistent with our study's findings and those of similar research.^2,11^ This is likely due to the elevation in intra-abdominal pressure, pelvic venous congestion, and potential trauma during childbirth.^2^

The limitations of the study are involvement of patients from a single tertiary care hospital, and thereby the results cannot be generalized to the entire population. Also, the study could not establish a cause-effect relationship because of the cross sectional nature of the study design.

## CONCLUSIONS

The prevalence of haemorrhoids in our center was found to be higher as compared to similar studies done in Southeast Asian region but similar with studies done in other region
